# Low-Power Wireless Data Transfer System for Stimulation in an Intracortical Visual Prosthesis

**DOI:** 10.3390/s21030735

**Published:** 2021-01-22

**Authors:** Adedayo Omisakin, Rob M. C. Mestrom, Mark J. Bentum

**Affiliations:** Department of Electrical Engineering, Eindhoven University of Technology, 5600 MB Eindhoven, The Netherlands; R.M.C.mestrom@tue.nl (R.M.C.M.); m.j.bentum@tue.nl (M.J.B.)

**Keywords:** low-power communication, intracortical visual prosthesis, inductive link, phase shift keying, non-coherent digital demodulator

## Abstract

There is a growing interest to improve the quality of life of blind people. An implanted intracortical prosthesis could be the last resort in many cases of visual impairment. Technology at this moment is at a stage that implementation is at sight. Making the data communication to and from the implanted electrodes wireless is beneficial to avoid infection and to ease mobility. Here, we focus on the stimulation side, or downlink, for which we propose a low-power non-coherent digital demodulator on the implanted receiver. The experimentally demonstrated downlink is on a scaled-down version at a 1 MHz carrier frequency showing a data rate of 125 kbps. This provides proof of principle for the system with a 12 MHz carrier frequency and a data rate of 4 Mbps, which consumes under 1 mW at the receiver side in integrated circuit (IC) simulation. Due to its digital architecture, the system is easily adjustable to an ISM frequency band with its power consumption scaling linearly with the carrier frequency. The tested system uses off-the-shelf coils, which gave sufficient bandwidth, while staying within safe SAR limits. The digital receiver achieved a reduction in power consumption by skipping clock cycles of redundant bits. The system shows a promising pathway to a low-power wireless-enabled visual prosthesis.

## 1. Introduction

An implanted intracortical visual prosthesis is perhaps the only possible technological option for most cases of blindness. Stimulating the visual cortex can be the last resort when the visual pathway is damaged or inaccessible. Approximately 216 million people worldwide are visually impaired, 36 million are blind [1], and most would need an intracortical visual prosthesis to regain some (rudimentary) form of vision. An intracortical visual prosthesis system generally comprises an external camera, a pocket processor, implanted electrode arrays, and a feedback loop [2].

A very high-count integrated wired system (65,536 electrodes) for neural recording and stimulation was reported in [3]. A desirable aspect for the visual prosthesis is that the powering and communication to and from the implanted electrodes be done wirelessly, to avoid infections and to enable free movement [4]. Wireless enablement is part of the goal of the NESTOR project which has the aim to implant a high count of electrodes (~1000) [5], using 16 Blackrock Microsystems’ Utah arrays of 64 electrodes each [6]. The wireless-enabled visual prosthesis will need a communication link to send stimulation information to the implant (downlink), another link for retrieving recorded neural activity from the brain (uplink), and wireless powering of the implant. The uplink is needed for adaptation, monitoring, and calibration over time. This work focuses on the downlink part of the system. The wireless power transfer system is designed separately in another sub-project.

**Figure 1:**
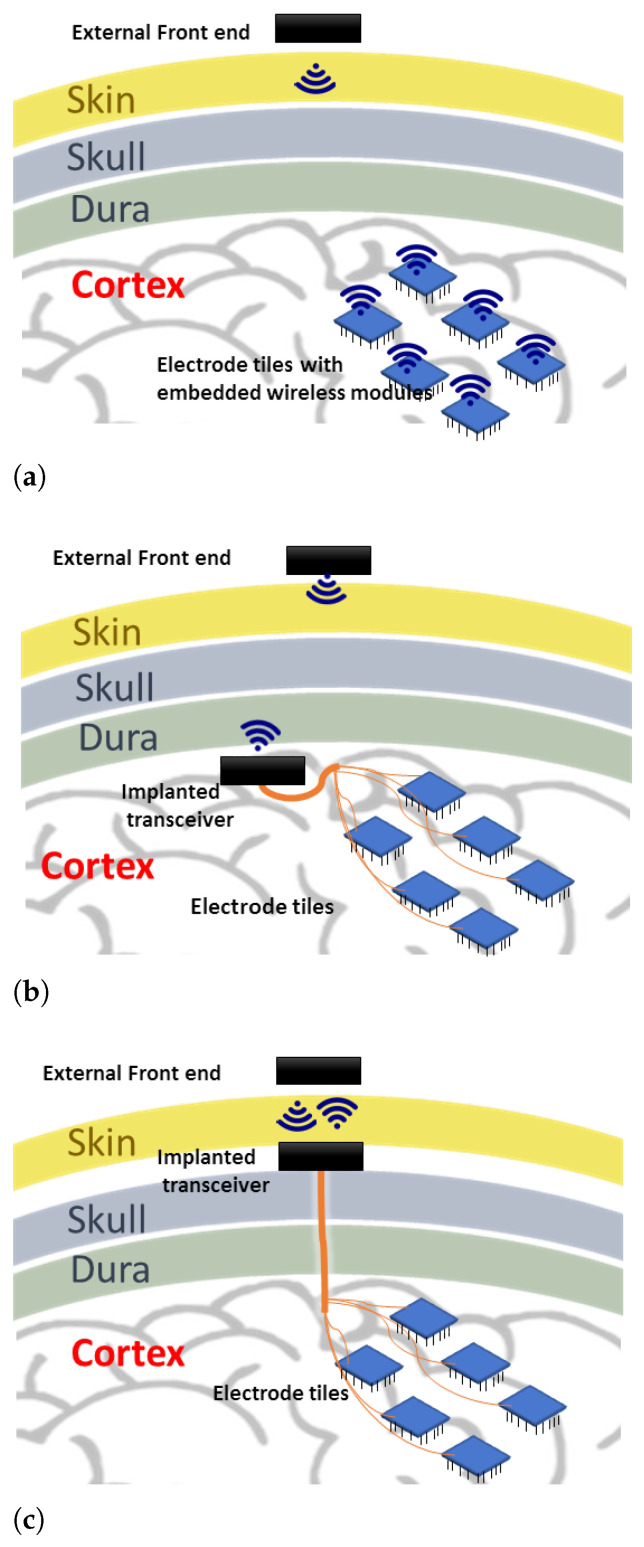
Various system layouts for the wireless enabled visual prosthesis. (**a**) place the implanted transceiver embedded into each electrode array; (**b**) use a central transceiver placed beneath the skull; (**c**) place the implanted transceiver beneath the skin.

There are a few possibilities in laying out this wireless system, which may directly impact the communication technology used for transmitting stimulation information. Figure 1 shows three possible layouts. The first option (Figure 1a) is to place the implanted transceiver embedded into each electrode array [7]. This form requires individual antennas/receive coils for each electrode array, which may face variable signal reception as the number of electrode array increases spatially. Another form (Figure 1b) is to use a central transceiver placed beneath the skull, but this space may not be convenient for the implanted transceiver if its components are bulky. The proposed form in this work (Figure 1c) is to place the implanted transceiver beneath the skin where more space is available, and a shorter distance to the external front end is present [8]. The challenge of this option is the possible micro-motion of the implanted connecting wires, which can be partly alleviated with better packaging and implantation.

Several general implanted medical links have been proposed for communication through the body [9,10,11,12,13,14]. Frequency shift keying (FSK) was investigated in [9]. Differential phase shift keying (DPSK) was used in [10]. In [11,12], binary phase shift keying (BPSK) was developed. Pulse delay modulation was implemented in [13]. A non-coherent analog BPSK demodulator was attempted in [14]. However, for the application to a visual implant, in which the overall system has to do simultaneous communication and powering and in which power consumption is an issue, the earlier works mentioned above are not suited. Instead, here we take an approach to arrive at a low-power system. Therefore, the requirements of developing the downlink for stimulation include low-power consumption <10 mW, data rates between 200 kbps and 4 Mbps, robustness to interference from the power carrier, and link security. These requirements led to take a low-power system approach which involves using low frequency <100 MHz, an inductive link for communication, phase shift keying (PSK), a nearly digital receiver, and non-coherent demodulation, as introduced in [15,16].

Furthermore, the system was simulated at circuit IC level showing 1 mW power consumption [17]. In this paper, we build on our previous work [15,17], with a focus on the following aspects: (1) to build a proof-of-principle demonstrator for the external transmitter and proposed implanted receiver; (2) to further reduce power consumption at the implanted receiver; (3) to test with off-the-shelf coils.

The remainder of this paper is structured as follows: Section 2 describes system considerations in terms of requirements and approach. Section 3 describes the proposed transmitter and the implanted receiver. Furthermore, we explain an approach for reducing the clock frequency by skipping periods in the non-coherent demodulation at the receiver. In Section 4, we describe the development of the demonstrator using off-the-shelf components. In Section 5, we present the results of the demonstrator, and in Section 6, we discuss the results in the context of future work: scaling for IC integration with the overall wireless-enabled visual prosthesis. Section 7 concludes the paper.

## 2. System Considerations

### 2.1. System Requirements

The overall system for the visual prosthesis will have many requirements for the system components various levels. We will now list the requirements related to the wireless communication system.

1. Low-power communication: based on power consumption reported in [18,19], the projected power consumption of the implant side of the 1000 electrodes visual prosthesis without wireless interface is on the order of 100 mW. This estimate includes neural recording electronics and the stimulation drivers. Considering the wireless power transfer, and battery constraints at the implant side, it is desired that the wireless system add no more than 10–30% extra power to the power budget at the implant side.
2. Data rate: if the stimulation pulses are to be transmitted in raw form, the total number of commands for a stimulation is 4 for a biphasic signal: (1) turn on the cathodal current; (2) turn it off; (3) turn on the anodal current; (4) turn it off. Each electrode needs to be refreshed at a rate of about 200 Hz for physiological reasons [20]. With this approach, the required data rate reaches a few Mbps. The implanted neurostimulation driver will take care of the waveform for stimulation. Only wireless communication to the driver is considered here. Therefore, from a video rate perspective coupled with a de-multiplexing scheme on the implant side, such as reported in [20], the minimum required communication data rate is 200 kbps (20 frames per seconds, 1000 electrodes per frame, and 10 bits per electrode). We aim for the 200 kbps.
3. Robustness to interference from power carrier: in the visual prosthesis, the implanted side will be powered wirelessly to reduce battery size constraints. The wireless power that reaches the implant side is of the order of 20 dBm [21], and the received data power can be in the order of −60 dBm. Thus, the power signal can be about 80 dB larger than the data signal. This power ratio may prove to be a challenge, especially when power transfer and communication take place simultaneously and at frequencies close together. Care must be taken that the frequencies are properly spaced to ensure that practical filters can be used without consuming a considerable amount of power.
4. Bit error rate: neural scientists desire that in the worst case, only one percent of the phosphene pixels is an error. If each electrode is encoded by 10 bits, then the worst case bit error rate target is $10^{-3}$. A much better bit error rate is preferred for robustness, the exact value is still unknown.
5. Security: with the rise of worldwide security breaches, measures must be taken to avoid hacking and re-writing of the brain. One of such measures could be taken at the physical layer by employing close range communication, which is inherently safer.

### 2.2. System Approach

**Figure 2:**
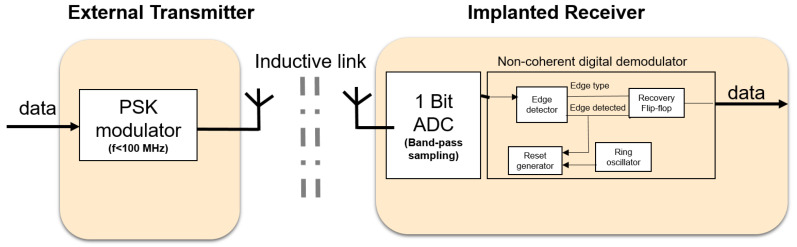
System architecture of the communication system for downlink.

The goal for the downlink is to have a low-power, robust communication system meeting the requirements stated in the previous section. Here we focus on the receiver side because this will be in the implant and therefore, poses the most challenging design. To consume less power, system components which can be power hungry such as phase locked loops (PLLs) and high-Q oscillators should be avoided where possible. In addition, high frequencies (above 100 MHz) should be avoided because they usually make devices consume more power and face more signal attenuation through the skin. This has motivated the use of alternatives to classical demodulation techniques, which use some of the power-hungry components mentioned earlier. As a result, Figure 2 shows the proposed downlink system blocks for the external transmitter and the implanted receiver. It contains the following features:

- Low frequency <100 MHz: the required downlink data rate is below 10 Mbps. Thus, modulating on carrier frequencies below 100 MHz will provide sufficient bandwidth. In addition, the electric and magnetic field will face minimal attenuation through the skin [22]. Lower frequencies also imply lower power consumption for the digital components.
- Inductive link: to eliminate the bulky antennas that are needed if low frequencies are used, an inductive link is proposed. The inductive link allows for short transmission ranges in the order of centimeters. This fits well with the application of the visual prosthesis, where transmission through a small layer of tissue (skin), at an implantation depth of 3 to 7 mm [23] is sufficient (see also Figure 1c) and security may be a concern.
- Phase shift keying (PSK): has a better theoretical bit error rate performance than other modulation schemes, such as amplitude shift keying (ASK) and frequency shift keying (FSK). Since the PSK modulation scheme is not sensitive to amplitude variations, it can easily cope with misalignment between transmitter and receiver coils, which has mainly effect on the signal amplitudes. Its transmitter is of similar complexity as in ASK. It is also spectrally more efficient than FSK which may require wideband inductive links [9].
- Bandpass sampling: at low frequencies (<100 MHz), the entire modulated signal (carrier and information) can be sampled or simply digitized and processed in the analog or digital domain.
- Non-coherent demodulation: using bandpass sampling at the receiver side, the entire received signal is sampled (digitized). It is possible to recover the information from the digitized received signal using a non-coherent digital technique. This avoids PLLs and allows for the use of low-power ring oscillators which have relatively large phase noise. The poor phase noise of the ring oscillator is not usually tolerated in classical demodulation techniques. Non-coherent analog demodulation for FSK and PSK generally uses envelope detection as part of the core demodulation process, and this has a poorer bit error rate (BER) performance than coherent demodulation [24]. However, this degradation in performance does not apply to the non-coherent digital demodulation proposed here as it does not use envelope detection. To overcome a weakness of our bandpass sampling approach, namely that it requires proper channel design, a coupled inductive link is used, to provide sufficient bandwidth for the channel.
- Differential encoding (optional): by using differential encoding and by matching the data rate to the power carrier frequency, we can improve the robustness to power carrier interference. This is based on the assumption that the power carrier interference is periodic and that each period of the signal will encounter the same interference when differentially encoded [25].

## 3. System Architecture

The communication link for transmitting the stimulation signals to the implanted electrodes (downlink), comprises of the external transmitter, the inductive link, channel and the implanted transceiver. Figure 2 shows the entire downlink system with its schematic sub-blocks. Frequencies <3 MHz or >25 MHz will be allocated for inductive powering for minimal skin losses, like the popular 125 kHz ISM band (<3 MHz) or the 401 MHz ISM band (>25 MHz) which gives room for filtering interference.

### 3.1. External Transmitter

**Figure 3:**
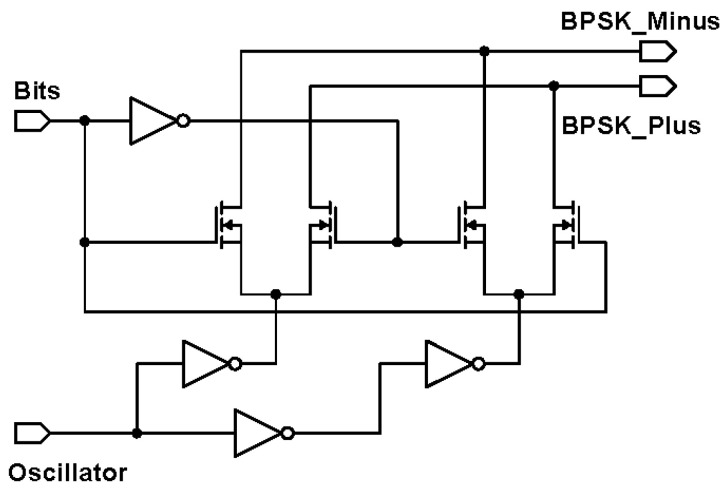
Circuit schematic of the binary phase shift keying (BPSK) mixer.

The external transmitter is essentially a binary phase shift keying (BPSK) transmitter. BPSK generally gives a good balance between power consumption and bit error rate performance [26]. To facilitate a future CMOS implementation, it comprises of an oscillator for generating the carrier frequency and a mixer for modulating the carrier signal. Since the intended carrier frequency ranges between 4 and 24 MHz to achieve sufficient data rate, the mixer can be low power, consuming about 1 mW in 180 μm CMOS technology [27]. The BPSK mixer designed is shown in Figure 3. The proposed mixer is comparable to the reported mixer in [27], with the exception that the transconductance current is not converted to a voltage level, by omitting the common-mode feedback structure. Two inverters act as switch pairs, switching between signal paths to generate the BPSK signal currents. Next, these BPSK currents of the external transmitter need to be passed to the implanted receiver through a suitable wireless link.

### 3.2. Implanted Receiver

At the receiver side, the entire signal is bandpass sampled to allow for digital demodulation. The bandpass sampling is essentially a 1-bit analog to digital converter (1-bit ADC) [14]. This can be implemented using a comparator. After this step, the resulting signal is then non-coherently digitally demodulated. The non-coherent digital demodulator is the central part of the receiver. These two parts will be described next.

#### 3.2.1. The 1-Bit ADC

From link circuit-model simulations, the received signal voltage level is expected to be in the range of 0.5 to 3 V. This may sometimes be lower than the digital logic level of the digital receiver, for example, if 1.8 V is used. Traditionally, low noise amplifiers are usually used as the first stage of an RF receiver. These may consume up to several milliwatts of power. However, in the context of the 5–10 mm inductive link, we propose a dynamic latch comparator as a low-power solution while delivering sufficient signal level during digitization.

#### 3.2.2. The Digital Demodulator

The non-coherent digital demodulator tries to detect if a ‘0’ or a ‘1’ was transmitted by detecting the type of edge it encounters in the digitized received modulated signal. The digitized modulated signal has a falling edge for the ‘0’ and a rising edge for the ‘1’. While detecting which type of edge is present, the subsystem must take care to avoid the transition point between symbols so that it is not detected as an edge type. The digital demodulator comprises mainly of an edge detector and a reset generator to reset the edge detector before the next symbol. The edge detector consists of a rising edge and falling edge flip-flop. With AND and OR logic gates, the type and timing of the occurring edges are determined. Through a D-flip-flop, the received bits are recovered from the edge type and edge detected. The detected edge is delayed to ensure alignment before entering the recovery D-flip-flop.

### 3.3. Inductive Link Design

**Figure 4:**
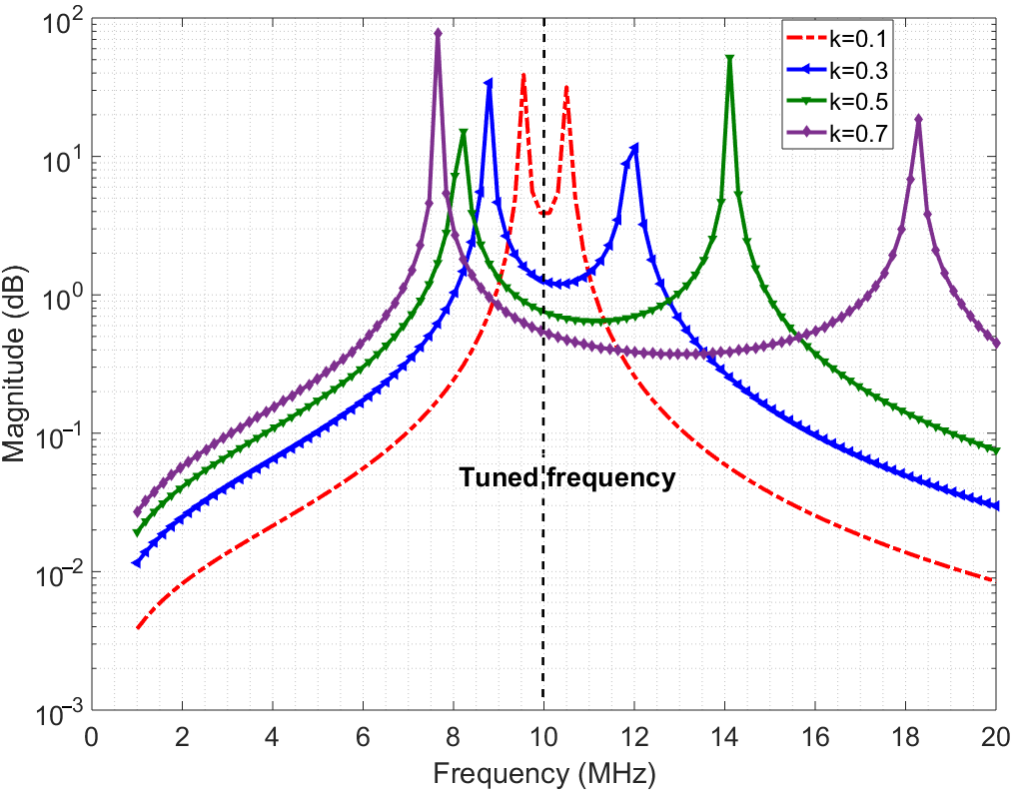
Ideal channel response.

The required data rate for the downlink ranges from 0.2 Mbps to 4 Mbps for the wireless enabled visual prosthesis [15]. With the proposed system layout of placing the implant side of the wireless module just beneath the skin, the distance from beneath the skin to above the skin is below 10 mm [23]. An inductive link is the most suited type of link because of the low attenuation of magnetic fields by the skin tissue and the compactness of the coils [16]. To keep power consumption low, the carrier frequency is chosen to be below 20 MHz. Achieving sufficient bandwidth for the desired data rate can be challenging, especially in inductive links. This is generally attempted by greatly reducing the quality factor of the coils which leads to more power dissipation [11]. The transition region of the PSK modulated signal can be distorted if the transmission of the inductive system is not flat enough in the passband. Creating such a (relatively) flat band can still be done without lowering the quality factor of the coils, by making the transmit and receive coils resonate at the same frequency. Furthermore, when the mutual coupling between the coils is high enough, the resulting coupled response gives two resonance peaks away from the resonance frequency of the individual coils, thereby creating a ‘well’ between peaks [28]. Figure 4 shows the ideal channel response for three values of the coupling factor. This well has a relatively flat passband to fit the data rate bandwidth. Empirically, it was found that the bandwidth is proportional to the coupling factor when the inductance is high enough (tens of microhenries), when the coupling factor is in range of 0.1–0.4, with a carrier frequency range of 0.1–30 MHz, and when the ohmic resistances of the coils are in the order of a few ohms [17]. When coupling factor is >0.4, the bandwidth is also proportional but with a slightly higher factor and mid-point.

### 3.4. Reducing Receiver Clock Frequency

The role of the reset generator module in the implanted receiver (see Figure 2) is to provide a reset signal to reset the edge detector at a time after the detection of the current edge t=0. This needs to be done at a time $0.5T_{psk}<t<T_{psk}$ to avoid the transition point between symbols, where $T_{psk}$ is the period of the carrier signal. To achieve this, it uses an asynchronous counter to count from a time of current edge detection t=0 to a time $0.5T_{psk}<t<T_{psk}$. We determine the frequency of the clock, which is essentially the frequency of the oscillator, as follows. With a count-up number *N*, the following inequalities must be satisfied. To reset after the transition point between carrier symbols, we have:

(1) $$\left(N-1\right)T_{osc}>0.5T_{psk},$$

and to ensure that there is no reset after the next edge, we get:

(2) $$NT_{osc}<T_{psk},$$

Therefore, the range for the clock frequency is given by:

(3) $$Nf_{psk}<f_{osc}<2\left(N-1\right)f_{psk},$$

where $f_{psk}=\frac{1}{T_{psk}}$ is the carrier frequency and $f_{osc}$ is the oscillator frequency. A practical reset timing constraint relates to the presence of realistic inductive links, in which the phase transition is sometimes not instantaneous. For flexibility in the reset timing, we write in a more general form:

(4) $$\frac{N}{c_{upper}}f_{psk}<f_{osc}<\frac{N-1}{c_{lower}}f_{psk},$$

where $c_{upper}$ and $c_{lower}$ are the set limits on the reset timing. It is observed that a higher count-up number *N* is needed if the range is to be made finer. This implies higher clock frequency.

The power consumption of the digital demodulator is proportional to the clock frequency. Since the carrier-to data ratio is below one due to bandwidth limitations, in the digitized received signal, there will be repeated bits, since more than one cycle of the carrier-waveform will represent a data symbol [17]. The clock frequency can be reduced by skipping a few periods *p* during the non-coherent demodulation. The reduction in clock frequency, in turn, will reduce power consumption. The carrier-to-frequency ratio is inversely proportional to the number of bits that will be repeated in the digitized received signal. For reducing the receiver clock frequency by skipping *p* periods, we get modified versions of Equations (1) and (2):

(5) $$\left(N-1\right)T_{osc}>\left(c_{lower}+p\right)T_{psk},$$

and

(6) $$NT_{osc}<\left(c_{upper}+p\right)T_{psk}.$$

The resulting clock frequency is given by:

(7) $$\frac{N}{c_{upper}+p}f_{psk}<f_{osc}<\frac{N-1}{c_{lower}+p}f_{psk}.$$

For instance, circuit simulation shows that at a data rate of 1.25 Mbps, a carrier frequency of 10 MHz and count-up number of N=16, the clock frequency of the digital demodulator can be reduced from 23 MHz to 5.8 MHz when p=2 periods are skipped. This reduces the power consumption from 0.29 mW to 0.15 mW for the non-coherent digital demodulator sub-block. Although generally, the overall power consumption is technology dependent, there is always a reduction in power consumption brought about by reducing the clock frequency by skipping redundant bits.

## 4. Experimental Demonstrator

For proof of principle of the downlink architecture, the system is built using off-the-shelf components and electronics. A scaled-down carrier frequency of 0.5–2 MHz and data rate of about 200 kbps is used for easy implementation on a breadboard, while still allowing for experimental investigation of key elements of the system, as described in the previous section. The demonstrator is not yet biocompatible. However, once the system has been implemented on an IC, an approach as described in [4] can be used to achieve biocompatibility. The following subsections present the experimental demonstrator in detail.

### 4.1. External Transmitter

**Figure 5:**
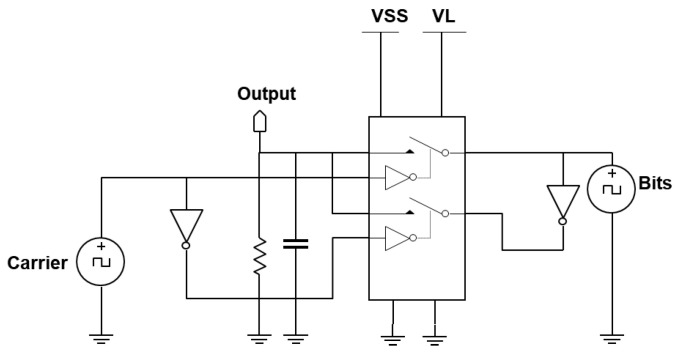
Circuit schematic of the emulated BPSK transmitter.

For the experimental demonstrator setup, the external transmitter, which is a BPSK transmitter, is emulated using two single pole single throw switches (SPST). Figure 5 shows the circuit schematic of the external transmitter demonstrator. To achieve BPSK functionality, the inputs into the second switch, which are the carrier signal and data bits, are inverted. The outputs of both switches are connected. Together, the switches act as a switch pair to form the BPSK modulated signals. The DC component is filtered using a high-pass filter. The two SPST switches are in a single component: the DG411LE from Vishay Siliconix Pennsylvania, USA. The inverters are SN74F04 from Texas Instruments, Texas, USA. By-pass capacitors of 0.1 μF were used to isolate the power supply of each sub-IC.

### 4.2. Practical Inductive Link

**Figure 6:**
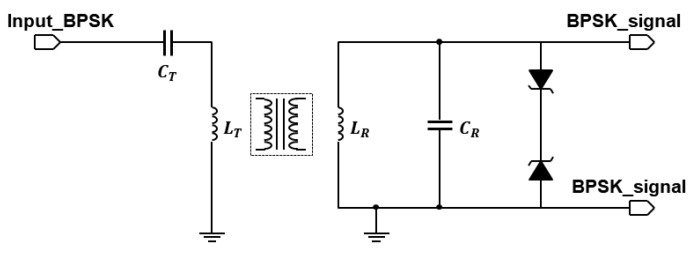
Inductive link schematic.

An extensive description of the design approach for the inductive link was carried out in [17]. Here, we focus on demonstrating the inductive link channel, for which we use off-the-shelf coils of moderate size with ferrite backing. These coils are initially not intended for data transfer, but their self-resonance frequency is above 15 MHz, making them suitable for the low-frequency demonstrator. We used a pair of 12 μH coils on a 48 mm by 32 mm ferrite plate for the transmit and receive coils. Alternatively, another pair of 10 μH coils on a 37 mm by 37 mm ferrite plate is used. Both sets of coils are from Wurth Electronics, Niedernhall, Germany. Ceramic capacitors of 2.2 nH are used to tune both the transmit coil and the receive coil to resonate at 1 MHz. Zener diodes are used to limit the maximum voltage to protect the comparator. Figure 6 shows the circuit schematic of this implementation.

### 4.3. Implanted Receiver

**Figure 7:**
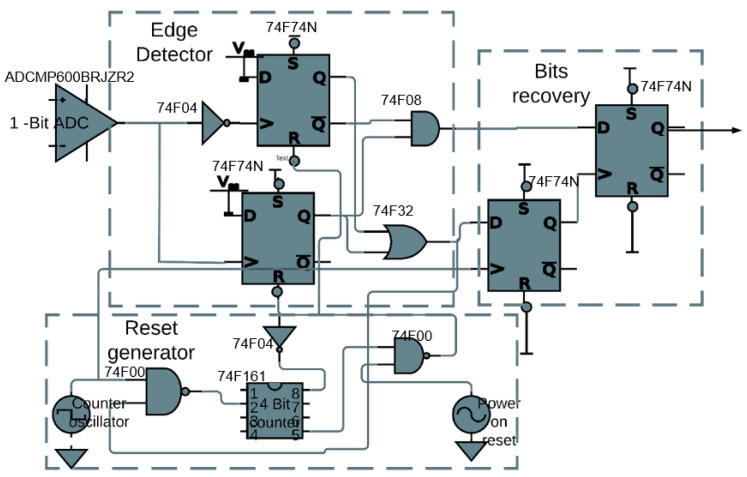
Downlink receiver demonstrator: schematic.

**Figure 8:**
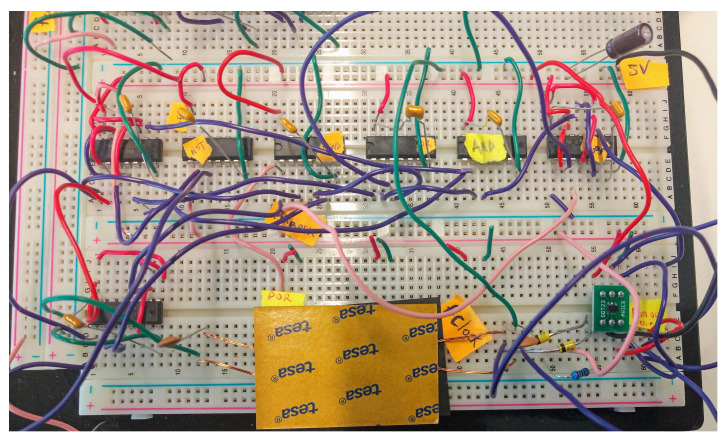
Downlink receiver demonstrator: realization.

Figure 7 shows the schematic of the circuit board implementation of the proposed implanted receiver. The ADCMP600BRJZR2 from Analog Devices, Massachusetts, USA. acted as the 1-bit ADC. The 74F74N dual D flip-flops along with the 74F08 AND gate and the 74F32 OR gate are used to construct the edge detector were from Analog Devices, Massachusetts, USA. The 74F04 NOT gate is inserted before the clock input to make it a negative edge. For the reset generator, the 74F161 4-bit counter is used from Analog Devices, Massachusetts, USA. In recovering the actual bits from the edge detector, two 74F74N flip-flops are used, one for alignment and the other for the recovery of the bits. By-pass capacitors of 0.1 μF are used (not depicted in Figure 7) to isolate the power supply of each IC. Figure 8 shows a picture of the implementation on a breadboard.

## 5. Results

With the demonstrator described in Section 4, the coupled coils are tested on open-loop voltage to determine the k-factor, which has an impact on the channel response. Next, the channel response is characterized by transmitting a sine wave and sweeping its frequency. Finally, data transfer is validated by sending the bits to the receiver, where they are recovered. The following sub-sections present these results, respectively.

### 5.1. Open-Loop Voltage on the Inductive Link

To determine the coupling factor between the transmit and receive coils separated by a certain distance, an open-loop voltage test was carried out: the coupling factor is found by determining the output voltage of the receiver coil divided by the input voltage of the receiver coil without any tuning capacitor. The input voltage has a source resistance of around 50 Ω. While estimating the coupling factor, the source resistance was corrected for. Layers of foam about 2.5 mm/layer were used to separate the transmit and receive coils to mimic the skin as in Figure 1. Below 100 MHz, the effect of skin on magnetic fields is negligible [22], so results with foam are expected to be almost the same as with skin tissue. Table 1 shows the received voltage value for 2 sets of coils at an input amplitude of 5 V. At 7.5 mm separation distance, the coupling-factor was slightly above 0.3 which still results in sufficient bandwidth for communication through the skin.

### 5.2. Inductive Link Response

**Figure 9:**
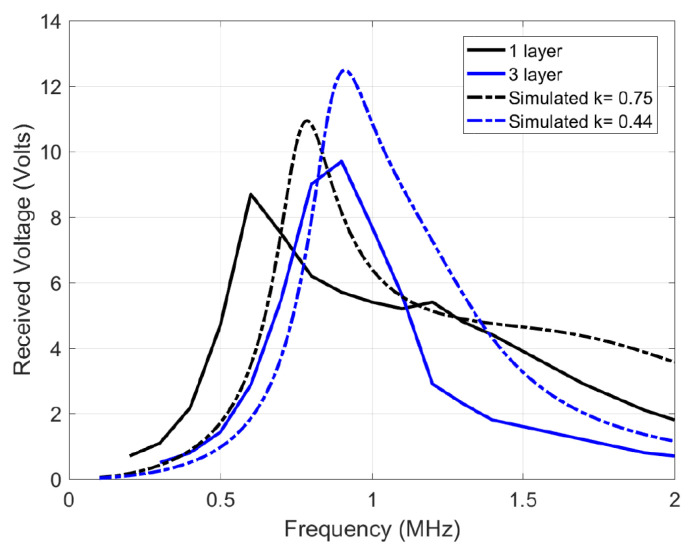
Channel response of the 12 μH transmit and receive coils, tuned individually to 1 MHz using 2.2 nF capacitors.

**Figure 10:**
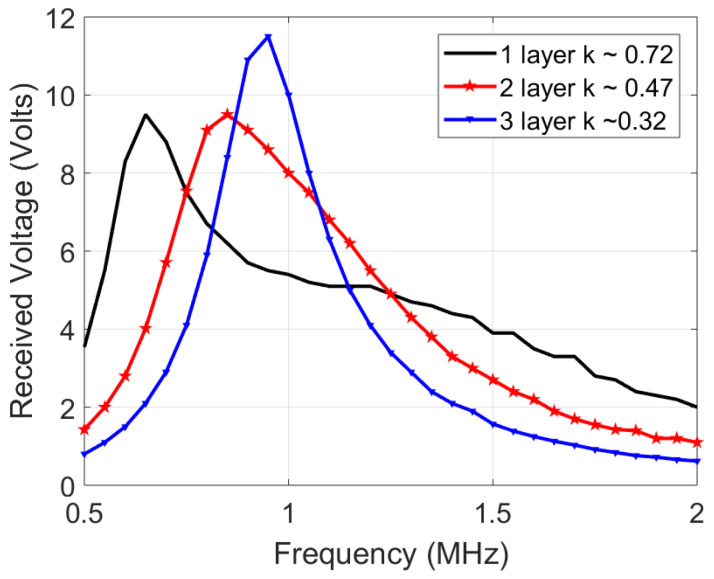
Channel response of the 10 μH transmit and receive coils, tuned individually to 1 MHz using 2.2 nF capacitors.

In estimating the channel response, an input single sine wave is swept in frequency, and its amplitude voltage at the receiver coil is recorded. However, one significant deviation is that the source is not resistance-free (unlike Figure 4), but has a 50 Ω impedance which decreases quality factor and the peaks, blurring out two peaks of the coupled response discussed earlier Section 3.3. Figure 9 shows the simulated response with a 50 Ω source impedance and measured results of the 12 μH coils. The slight deviation between the simulated and measured plots is due to measurement errors, deviation in component values and impedance differences. Figure 10 shows the channel response on the 10 μH coils at 3 different layers. As expected, the 50 Ω impedance decreases the quality factor, blurring the two resonance peaks. In addition, a smaller separation distance (less layers of foam) results in more bandwidth because of the higher coupling factor, which is similar to the ideal response in Figure 4. The similarity in bandwidth behavior between Figure 4, Figure 9 and Figure 10 indicate everything is in order for scaling up to the 12 MHz range. The peaks are compared other than the tuned frequency because they indicate the bandwidth for the data transfer available as explained in Section 3.3.

### 5.3. System with Inductive Link

**Figure 11:**
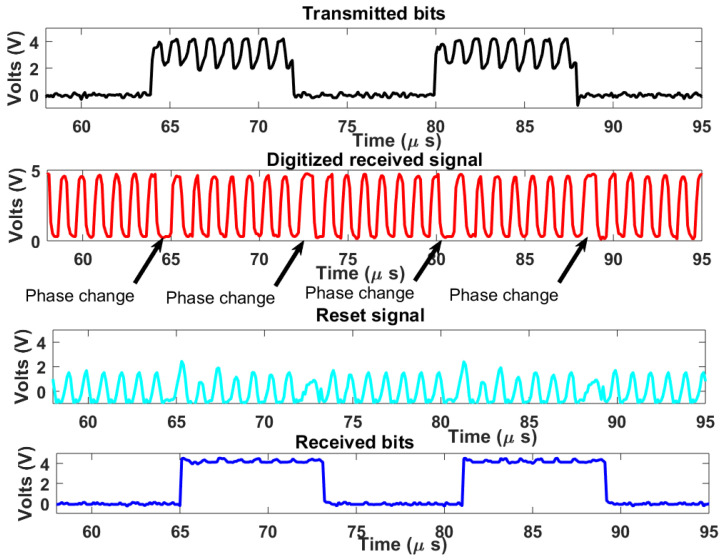
Measured signal results.

The complete downlink system demonstrator is tested on the breadboard setup. A carrier frequency of 1 MHz is used as well as a bitrate of 125 kHz. The clock of the digital receiver was set to $10*f_{psk}$ which is 10 MHz. In this case, a count-up number N=8 was used, which also satisfies the range constraint in Equation (7). Figure 11 shows the measured signal results. The transmitted bits had some carrier patterns due to imperfect isolation between sub-blocks. On the digitized received signal, phase changes due to bit type changes in PSK modulation can be seen in areas with a longer period. The digitized signal waveform also indicates that the desired bandwidth was achieved. The reset signal is also shown in Figure 11, every rise in the reset signal indicates a reset triggering by the reset generator for digital demodulation. The recovered bits are in agreement with the transmitted bits indicating successful demodulation with a typical harmless time lag arising from the demodulation process.

### 5.4. Overall Results

The system meets all the requirements for low-power communication: <1 mW; data rate: potential of 4 Mbps on full system scaling; Robustness to interference: 125 kHz could be used for power transfer; bit error rate: by flexible oscillation range; and security: by the near-field coupling to demodulate the signal. With transcutaneous communication, the inductive coupling using coils makes it secured physically from external intrusion.

## 6. Discussion

### 6.1. Scaling

A scaled-down carrier frequency of 1 MHz and data rate of about 125 kbps are used for easy implementation on circuit board for proof of principle. The data rate is sufficient for testing since the 125 kbps data rate is close to the lower bound of the requirements of the data rate for the final system. The system will be easily scaled up to operate at 12 MHz and at a higher data rate up to 4 MHz, as our IC simulation on CMOS 0.18 μm at 1.8 V has shown [17]. It is important to stress that the power consumption of the receiver scales linearly with frequency due to its predominantly digital circuits. Consuming below 1 mW at 12 MHz on IC simulation clearly indicates that tuning to a 6.7 or 13.56 MHz ISM band is easily possible although low-power communication via a 5-10 mm magnetically coupled (inductive) link is quite different from electromagnetic RF communication using antennas.

### 6.2. Comparison with Other Works

Table 2 compares our work with other reported related work. Our receiver is low-power compared to others. The 0.95 mW consumed by our receiver will even be much lower in more advanced CMOS technologies because of the predominantly digital circuits in our implementation compared to others. The receiver presented in [29] appears to be more energy-efficient and delivers a higher data rate, but in our application, the frequency used in [29] may coincide with the future uplink. Furthermore, our achieved data rate is sufficient for our application. It can be much more energy efficient by scaling the supply voltage and CMOS technology used since our architecture is predominantly digital. Compared to [30], our work is already more energy-efficient and has a higher data rate potential. Even higher data rate is possible than other works by merely scaling the carrier frequency, but a practical inductive link with sufficient bandwidth may be a limiting factor. In an IC implementation, the signals are expected to be less noisy as lengthy wires that cause interference will be eliminated. Then an accurate bit-error-rate (BER) measurement will be carried out. A BER better than $10^{-3}$ is acceptable and expected. In the worst case, this may cause a few data packets to be occasionally discarded since the application is in real-time.

### 6.3. Tissue and Safety

It is essential to mention the safety of the system in regards to the SAR limits and tissue heating. While the scaled proof of principle demonstrator in Section 4 does not focus on low-power consumption, the designed receiver on integrated circuit shows simulation results of consuming below 1 mW. With this, a theoretical maximum of 1 mW of power is transmitted via the skin for data communication. In [21], 1000 mW was transmitted in SAR simulations illustrating that 10 g averaged SAR levels of maximum 1.92 W/kg are obtained. This value is below the specified 2 W/kg safety level. Our system’s 1 mW through the skin data communication is very far below the SAR safety limit. In addition, regarding heat dissipation into the tissue by the implanted receiver, to prevent damage at the electrode–tissue interface, the maximum temperature increase in the cortex has to be smaller than 1 ${}^{{}^{\circ}}$C. This corresponds to a maximum power density of 0.8 mW/mm${}^{2}$ of exposed tissue area [18,34,35]. Our simulated implanted IC consumes below 1 mW in a 25 mm${}^{2}$ package bringing the power density to 0.04 mW/mm${}^{2}$, which is well below the safety limit.

## 7. Conclusions

The downlink for delivering stimulation data to a visual prosthesis was experimentally demonstrated. Commercial off-the-shelf coils were used, showing sufficient bandwidth. Power consumption at the implanted receiver was reduced by lowering the clock frequency by skipping redundant bits in the digitized received signal. The system meets the specified requirements.

Although the system was demonstrated on a scaled-down version on a circuit board, the experimental results show everything is in order from a system point of view for a more compact, integrated design, together with the uplink system in future. This will wirelessly enable the visual prosthesis in a low-power and robust way.

## Figures and Tables

**Table 1:** Open-loop voltage test to estimate the coupling factor.

**12 μH Coil [48 mm by 32 mm Ferrite Plate]**		
	Open-loop voltage	k-factor
1 layer [2.5 mm] 5 V input @ 1 MHz	3.30 V	0.80
3 layer [7.5 mm] 5 V input @ 1 MHz	1.90 V	0.46
**10 μH Coil [37 mm by 37 mm Ferrite Plate]**		
	Open-loop voltage	k-factor
1 layer [2.5 mm] 5 V input @ 1 MHz	3.30 V	0.80
3 layer [7.5 mm] 5 V input @ 1 MHz	1.27 V	0.31

**Table 2:** Comparison with other biomedical implants.

Reference	[14]	[30]	[29]	[31]	[32]	[33]	This Work
Comm, tech.	BPSK	BPSK	FSK	BPSK	OOK	ASK	BPSK
Freq. (MHz)	20	13.56	902–928	0.125	401–419	10	12
Datarate (Mbps)	2	1.12	8	0.01	0.01–2	1	0.1–4
Pow. cons. (mW)	6.2	0.7	0.6	-	-	-	0.95
Energy cons. per bit (μW/Mbps)	3100	625	75	-	-	-	238

## Data Availability

Data is contained within the article tables and figures and are available on request.

## Abbreviations

The following abbreviations are used in this manuscript:

ISM	Industrial, Scientific, and Medical
IC	Integrated Circuit
SAR	Specific Absorption Rate
NESTOR	NEuronal STimulation fOr Recovery of Function
BPSK	Binary Phase Shift Keying
PLL	Phase Locked Loop
FSK	Frequency Shift Keying
ASK	Amplitude Shift Keying
BER	Bit Error Rate
CMOS	Complementary Metal–Oxide–Semiconductor
SPST	Single Pole Single Throw
ADC	Analog to Digital Converter
